# Whole-genome sequencing of Tahe red deer (*Cervus hanglu yarkandensis*) reveals genetic diversity and selection signatures

**DOI:** 10.3389/fvets.2025.1642382

**Published:** 2025-08-21

**Authors:** Te Pi, Wenfeng Yi, Zengwei Mao, Zhihua Wang, Hao Sun, Shouqing Yan

**Affiliations:** College of Animal Science, Jilin University, Changchun, China

**Keywords:** genetic diversity, population structure, selection signature, Tahe red deer, whole-genome sequencing

## Abstract

The Tahe red deer (TRD), domesticated and artificially raised from wild Tarim red deer, is valued for its high-quality antlers and ability to survive tough desert conditions. Nowadays, the decline in the population of TRD has significantly impacted their genetic diversity, posing a serious threat to their conservation and utilization. However, information based on whole-genome sequencing data of TRD is scarce, and the mechanisms underlying adaptive characteristics remain poorly understood. Additionally, research on Tahe red deer holds great importance for elucidating the evolutionary history and adaptability of the genus *Cervus*. This study aimed to investigate the genetic diversity, population structure, and selection signals of TRD using whole-genome sequencing data. The results revealed that TRD exhibited high inbreeding level and relatively low genetic diversity, and that TRD had a closer relationship with *Cervus canadensis*. Using three methods, including the fixation index, nucleotide diversity and cross-population extended haplotype homozygosity, there were 573 genes annotated in 2,303 overlapping candidate selection regions such as *IL1R1, F13B, ARHGAP15, DCLK3, CACHD1, NDEL1,* and *UPF1*, most of which were associated with adaptation to a hot arid environment. In summary, this study offered genomic markers and candidate genes associated with these traits, providing valuable insights for improving future breeding strategies of TRD.

## 1 Introduction

Adaptation represents a continuous and long-term evolutionary process, during which beneficial alleles accumulate at gene loci (1). Species within the genus *Cervus* have evolved distinct survival strategies and physiological adaptations over time, shaped by various environmental factors such as climate and habitat types. Understanding the mechanisms by which populations or species respond to environmental changes is essential for the conservation of key species under the pressure of global climate change (2).

The genus *Cervus* with its diverse populations across China has long been extensively farmed for a variety of valuable products such as antler velvet and venison. The Tarim red deer found in the Tarim River basin, as an essential component of the local ecosystem, exhibits considerable biological adaptability under harsh desert environment (3). In recent decades, the fragmentation of the wild Tarim red deer’s habitat, caused by increased human activities and climate change, has led to a further decline in the wild population (4). Accordingly, China has classified the wild Tarim red deer as a first-class nationally protected wild animal and implemented a series of conservation measures to protect this endangered species. The Tahe red deer (TRD) was domesticated and artificially raised from the wild Tarim red deer (*Cervus hanglu yarkandensis*) by the Second Agricultural Division of Xinjiang Construction Corps since the 1950s (5). As an artificially bred population with significant economic and ecological value, TRD is highly regarded for its high antler yield and remarkable ability to cope with arid conditions. Due to a lack of awareness and the impact of market forces, the number of TRD has witnessed a sharp decline, with the current stock being less than 10,000, posing a serious threat to their genetic diversity (6).

Currently, studies on the genetic diversity of TRD mainly focuses on the sequences from Y chromosome and mitochondrion, and the results indicates an imbalance with high haplotype diversity and low nucleotide diversity (6). Analyses on the phylogenetic relationships about the Tarim red deer have primarily relied on mitochondrial genomes and markers (6–8). Mitochondrial control region analyses have identified two distinct lineages: the Western lineage including red deer (*Cervus elaphus*) and the Tarim red deer, and the Eastern lineage comprising wapiti (*Cervus canadensis*) and sika deer (*Cervus nippon*) (7). However, studies based on cytochrome b gene and D-loop region have identified the red deer, the Tarim red deer, and wapiti as three individual species (6–9). Studies based on mitochondrial DNA and microsatellite markers have not reached a concordant conclusion on the phylogeny of *Cervus*. Compared with mitochondrial DNA and microsatellite markers, nuclear DNA data can more accurately reveal genetic differences between breeds, making it widely used to evaluate genetic diversity and detect selective sweeps (10). Undoubtedly, the assessment of genetic diversity and population structure based on genomic information will be essential for the better breeding management and sustainable use of genetic resources.

TRD has unique adaptability that makes it an ideal subject for studying how animals cope with extreme conditions (11). The antler velvet with high yield serves as a vital ingredient in traditional Chinese medicines and health products (6). Given that the valuable genetic resource is severely depleted, rational conservation and utilization are urgent. In addition, the study of TRD is of great significance for understanding the evolution and adaptability of the genus *Cervus*. Presently, information on genomic diversity of TRD based on the whole genomic sequences is scarce and the publicly available genomic data remain limited. This study assessed the genetic diversity in TRD and identified candidate genes associated with adaptation to hot arid environments using whole-genome data from 17 TRD individuals, thereby providing valuable insights into important genetic variations and the conservation of genetic resource.

## 2 Materials and methods

### 2.1 Sample selection

Antler slices of 17 TRD were purchased as the commercial products from the farm located in Korla City, Xinjiang Uygur Autonomous Region of China (Supplementary Figure S1). Genomic DNA was extracted for WGS using the EasyPure Genomic DNA Kit (TransGen Biotech). DNBSEQ-T7 was used at the Novogene Bioinformatics Institute company (Beijing, China) to generate 2 × 150 bp paired-end read data for each individual (Supplementary Table S1). Additionally, to enhance our understanding of the genomic genetic diversity and selection signals in TRD, WGS data for 27 published individuals including *Cervus elaphus hispanicus* (ERD, *n* = 14) and *Cervus canadensis nelsoni* (CRD, *n* = 13) were acquired from the Sequence Read Archive.^1^

### 2.2 Reads mapping and quality control

Raw data were filtered using FASTP v 0.20.1 software (12). Burrows-Wheeler Aligner (BWA) software (v0.7.13) was used for mapping all cleaned data to the red deer reference genome (GCF_910594005.1, mCerEla1.1) using “bwa mem” parameters (13). The aligned BAM files were sorted using SAMtools v1.19 (14). Subsequently, the Picard MarkDuplicates tool (v1.115)^2^ was employed to mark duplicate reads from each alignment. After sorting reads, variants were called using the Genome Analysis Toolkit (GATK v4.1.4) and filtered with GATK’s “VariantFiltration” module (15). The hard filters were applied to the raw SNPs according to the criteria as follows: QD < 2.0 || FS > 60.0 || MQ < 40.0 || SOR > 3.0 || MQRankSum < −12.5 || ReadPosRankSum < −8.0. Quality control was then performed using PLINK (v1.9) with the parameters “--geno 0.05 --mind 0.1,” ensuring that each locus retained a minimum of 3 alleles, and biallelic loci on autosomes were acquired using BCFtools (v1.8) (14, 16). The remaining SNPs were subsequently used for further analysis. Based on the mCerEla1.1 reference genome annotation file, the variants after quality control in TRD were annotated using SnpEff software (v5.1d) (17).

### 2.3 Genetic diversity analyses

To assess genetic diversity, expected heterozygosity (*H*_E_), observed heterozygosity (*H*_O_), nucleotide diversity (*pi*) and the runs of homozygosity (ROH) were estimated. PLINK (v1.9) software was used to calculate *H*_O_ and *H*_E_ with the option “—hardy” (16). VCFtools software (v0.1.16) was employed to assess *pi* with the parameters “--window-pi 20,000 --window-pi-step 10,000” (18). To evaluate the inbreeding degree, the number and length of ROH fragments were calculated using VCFtools with the “—LROH” command (18). After that, the runs of homozygosity-based inbreeding coefficient (*F*_ROH_) were calculated as the total length of ROH fragments divided by the length of the autosomes. PopLDdecay (v3.42) was applied to assess the degree of linkage disequilibrium decay (LD) of each population by calculating the pairwise SNP correlation coefficients (r^2^) with the parameter “-MaxDist 1,000” (19).

### 2.4 Population structure analysis

The NJ method was applied to construct a tree based on Nei genetic distance using VCF2Dis (v1.47) and visualized with the Splitstree software (20–22). Sites with high linkage disequilibrium (LD) were removed using PLINK with the parameter “-indep-pairwise 50 25 0.2” and the remaining SNPs were used for the following population structure analysis (16). Principal component analysis (PCA) was performed using GCTA software (v1.92.3) with the parameter “grm” and plotted in the R package ggplot2 (23, 24). Admixture analyses were conducted using ADMIXTURE software (v1.3.0) with the parameters “admixture -cv” to infer ancestral populations in our dataset (25). Cross-validation error values for clustering at K = 2 to 4 were also calculated. BEAGLE (v5.4) software was employed to first phase the data file and impute missing genotypes (26, 27). Haplotypes were then inferred with Refine IBD, using a 40-SNP sliding window, a 1.5 cM minimum haplotype length, a 0.15 cM trimming threshold, and a LOD score of 1 (28). The counts of haplotypes shared between populations were visualized with the circlize package in R (29).

### 2.5 Genome-wide scanning for selection signatures

To identify the candidate regions and genes associated with the unique traits of TRD compared to CRD and ERD, three analytical methods were performed: the fixation index (*F*_ST_), *pi* and cross-population extended haplotype homozygosity (XP-EHH). Selection signal values were computed based on a 20 kb sliding window. The *pi* and *F*_ST_ values were calculated using VCFtools (v0.1.16) with the parameters “--window-pi 20,000 --window-pi-step 10,000” and “--fst-window-size 20,000 --fst-window-step 10,000,” respectively (18). Additionally, haplotype phasing was conducted using BEAGLE (v5.4), and the phased data were then used to estimate XP-EHH (30). To detect the positive selection signatures between TRD and other populations, XP-EHH was calculated using selscan (v1.1), and the results were subsequently normalized using the “-norm” module of selscan for each 20 kb region (31). To reduce false positives, only the top 5% genomic windows from the three methods were selected for further analysis. The genomic regions identified by all three methods were intersected using BEDtools (v2.30.0) with the “intersect” parameters to determine the potential candidate regions of selection (32). Functional annotations of the identified regions were carried out using SnpEff software (v5.1d). To gain a better understanding of the identified candidate genes, Gene Ontology (GO) and Kyoto Encyclopedia of Gene and Genomes (KEGG) pathway enrichment analyses were performed using KOBAS, with cow (*Bos taurus*) genome selected as ortholog (33). Only when the *p*-value was less than 0.05 were the pathways considered to be significantly enriched.

## 3 Results and discussion

### 3.1 SNP genotyping and annotation of TRD

In total, 675.51 Gb of raw data from 17 TRD individuals was obtained through the whole-genome resequencing (Supplementary Table S1). After the filters, 671.74 Gb of clean data were retained. The sequencing quality metrics indicated an average Q20 of 99.49%, an average Q30 of 98.08%, a GC content of 43.37%, mapping rates between 99.23 and 99.88%, and an average sequencing depth of 13.44×. Furthermore, 12,086,946 high-quality autosomal bi-allelic SNPs were detected in the SNP dataset of 17 TRD after quality control, of which no individuals were excluded based on the “mind 0.1” criterion. Among the identified variants, there were 8,819,706 transition SNPs (T_S_) and 3,267,240 transversion SNPs (T_V_), leading to a T_S_/T_V_ ratio of 2.70. The least number of SNPs was observed in chromosome 26, while the highest number of SNPs was found in chromosome 30, with an average variants frequency of 4.47 SNPs/Kb (Supplementary Table S2). A detailed SnpEff annotation showed that most SNPs were found in intergenic regions (47.26%) and intronic regions (38.42%), with only 1.01% located in exonic regions (Supplementary Table S3). Among the 121,553 exonic SNPs identified, there were 66,662 synonymous variants, 54,167 missense variants, and 724 nonsense variants.

### 3.2 Genetic diversity analysis

**Figure 1:**
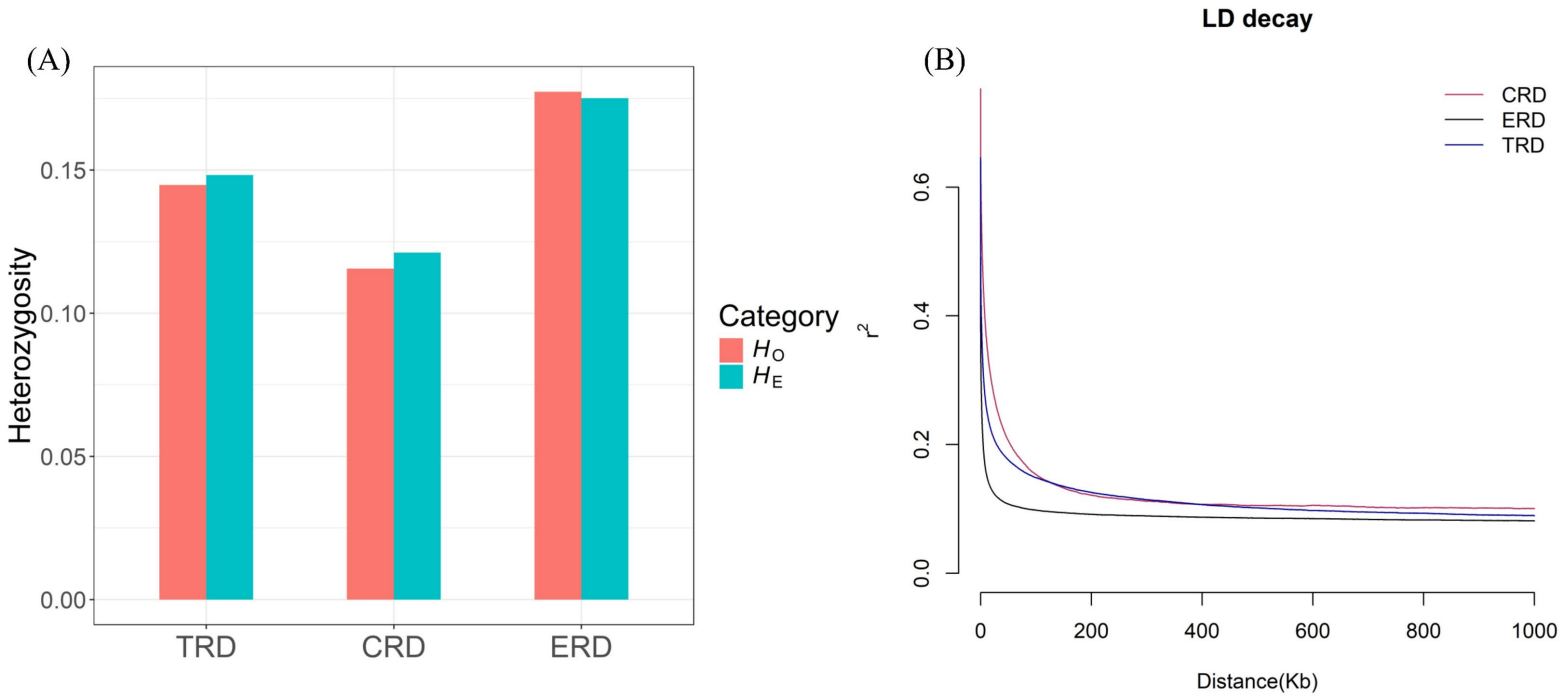
Genetic diversity of 3 populations. **(A)** Distribution of expected heterozygosity (*H*_E_) and observed heterozygosity (*H*_O_) in each population. **(B)** Decay of linkage disequilibrium (LD) on autosomes was estimated from 3 populations.

The average values of *H*_O_, *H*_E_, ROH and *pi* were computed to evaluate the genetic diversity of all populations (Supplementary Table S4). The results indicated that the pi value ranged from 0.0013 to 0.0020. Among them, TRD (0.0016) was higher than CRD (0.0013) but lower than ERD (0.0020). Besides, the *H*_O_ and *H*_E_ of TRD was between CRD and ERD (Figure 1A). Consistent with the research on the genetic diversity of TRD based on mitochondrial sequences and the Y chromosome, the results also indicated a low level of nucleotide diversity (6). The *F*_ROH_ value of TRD was 0.2729, which was much higher compared to ERD. As for LD decay, the r^2^ values declined sharply as genomic distance increased in all populations, with the most rapid decline occurring within the first 50 kb (Figure 1B). This indicated that there had been a significant amount of recombination in the recent history of TRD. When the distance between markers exceeded the first 50 kb, the results indicated that TRD had a relatively high LD level, whereas ERD had the lowest. These values suggested that TRD may have relatively low genetic polymorphism and inbreeding within the population, which reminds us that rational conservation measures and breeding plans should be established to conserve the genetic resource.

### 3.3 Population structure analysis

**Figure 2:**
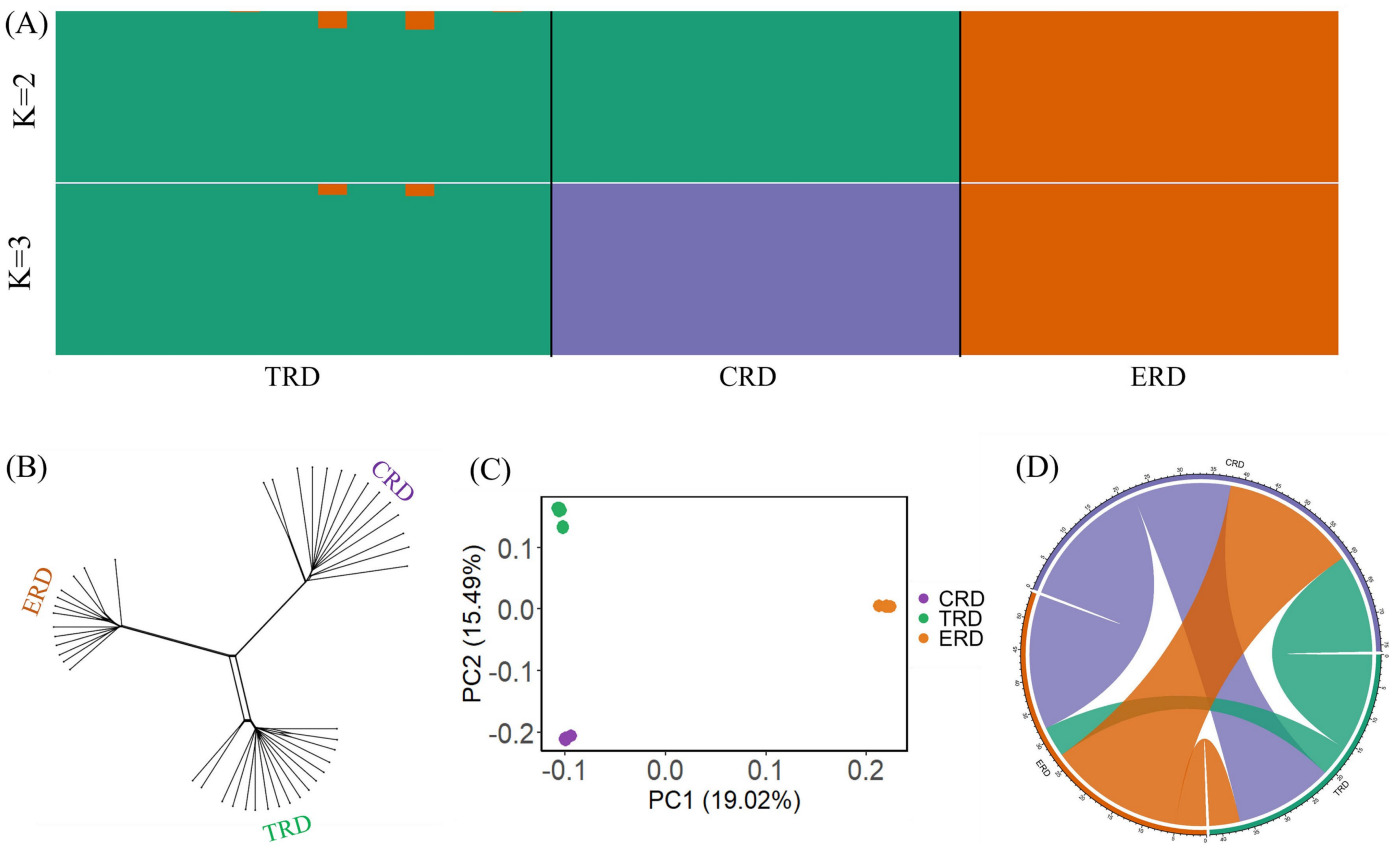
Population structure and relationships of TRD with other populations. **(A)** Result of admixture (*K* = 2 and 3). **(B)** Neighbor network constructed from Nei. **(C)** Principal component analysis (PCA). **(D)** Circos plot of the number of shared haplotypes.

To investigate the genetic distances among different populations, phylogenetic analysis was performed on 44 individuals representing three populations. It can be observed from the Neighbor-Net network that three populations formed clearly distinct clusters from one another and TRD was more closely related to CRD than to ERD, which was different from the previous research finding on the basis of comparative genomics (Figure 2B). The 2,506,148 SNPs after linkage pruning were used for PCA and admixture analysis to further investigate the cluster patterns between TRD with other populations (Figure 2A). The lowest cross-validation error value was observed at *K* = 3, as the hypothetical ancestral groups were evaluated for K values between 2 and 3. When *K* = 2, these different populations can be genetically divided into two groups: one group consisted of ERD, while the other group included CRD and TRD. When *K* = 3, TRD showed distinct admixture component proportions compared to the CRD. In PCA analysis, the first principal component (PC1) accounted for 19.02% of the variation in the genomic data, while the second principal component (PC2) explained 15.49% (Figure 2C). The PCA result showed that TRD exhibited a closer genetic relationship with CRD compared to ERD, which was consistent with Neighbor-Net network and ADMIXTURE results. The haplotype exchange analysis between populations identifies more pronounced haplotype exchange between CRD and TRD, while revealing reduced IBD sharing between TRD and ERD (Figure 2D). Moreover, TRD was genetically closer to CRD than to ERD. In addition, mitochondrial phylogenetic tree indicated that TRD had a closer genetic relationship with ERD than CRD. Phylogenetic analysis based on comparative genomics and mitogenomes were inconsistent with the nuclear DNA phylogeny, probably due to incomplete lineage sorting or genetic introgression (10, 34). Whole genomes would provide more accurate evidence in this respect because nuclear DNA data contains a greater quantity of genetic markers. One might interpret it as the Tarim red deer differentiated relatively early due to either an extreme cold climate or human activities, evolved independently in an isolated environment, and had a distant genetic relationship with other CRD populations (4). Given the individuals selected for this study were domesticated, the results of population structure and phylogenetic relationships may be influenced by the size and source of the sampled populations. Therefore, samples from a larger number of TRD and wild Tarim red deer should be included to verify whether this pattern remains consistent.

### 3.4 Selection signature analysis

**Figure 3:**
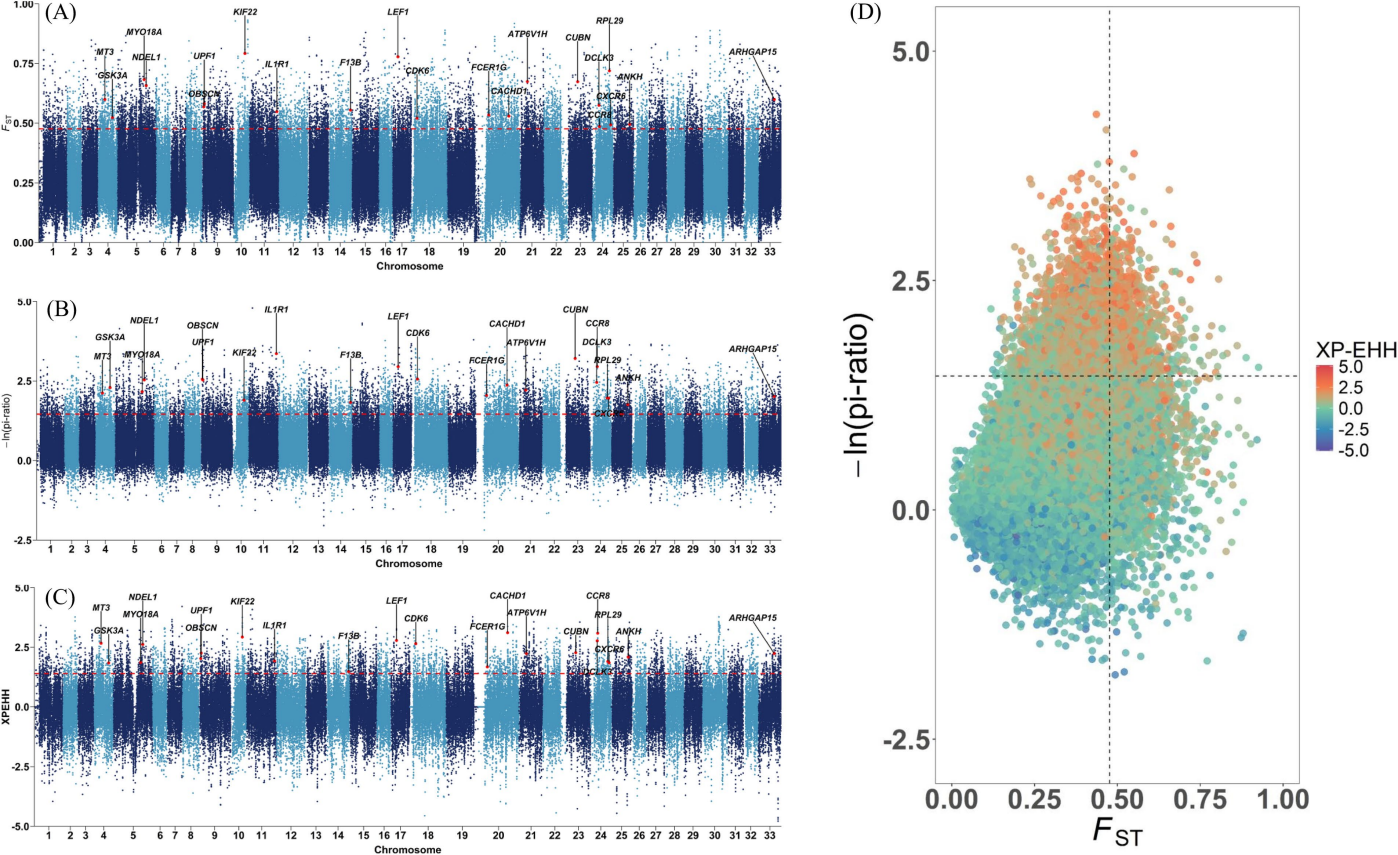
Manhattan plots of selection signature between TRD and other populations. The dashed line is the 5 percent threshold. **(A)** The fixation index (*F*_ST_). **(B)** Nucleotide diversity (*pi*). **(C)** Cross-population extended haplotype homozygosity (XP-EHH). **(D)** Signal intersection analysis of *pi*, *F*_ST_, and XP-EHH.

Two wild populations were selected as the reference populations, including the European red deer (ERD), which primarily inhabit mountain forests with a Mediterranean climate, and the North American Elk (CRD), which live in forests dominated by pine trees with distinct seasonal changes. The Tarim red deer have evolved to cope with the heat and arid desert environment, but the mechanisms underlying their adaptive features remain poorly understood. In the selection signature analyses, there were 11,673 regions, 12,091 regions and 6,441 regions ascertained through *pi*, *F*_ST_ and XP-EHH methods in the top 5%, respectively. For example, *CACHD1*, *CPLX1*, *DIS3L2*, *MAGI2*, *MYH10*, *NCAM2*, *NDEL1*, *POC1A*, *SUPT3H*, *TWF2*, and *UPF1*, which have high selective sweeps identified by both *pi* and *F*_ST_ methods in TRD, have also been identified as candidate genes in sheep breeds adapting to desert environments (35). Only the top 5% of windows identified by all three methods were used to pinpoint candidate regions and genes. In total, 2,303 candidate selection regions were identified including 573 genes likely subject to strong selection in TRD. The detailed information regarding these regions was depicted in Figure 3 and documented in Supplementary Tables S5–S8. Study indicates that *ARHGAP15*, which regulates various biological processes such as cytoskeletal dynamics and cell movement, is linked to physiological traits related to tropical adaptation in Zaobei Beef cattle (Table 1) (36). *F13B*, a gene strongly selected in TRD, has also been pinpointed as a candidate gene for the desert adaptation of Bactrian camels (37). Meanwhile, *DCLK3* (doublecortin like kinase 3), characterized by its biased expression in testis and brain, might be involved in the heat stress and essential for improving thermoregulation in sika deer (38). Several candidate genes, including *MYO18A*, *FCER1G*, *IL10RB*, *CCR8,* and *CXCR6*, are connected to the immune system and environmental adaptation, which might be useful for the survival of the Tarim red deer in the Tarim Basin (39–42). *ANKH* regulates bone formation and skeletal development by inhibiting the mineralization process through the encoding of an inorganic pyrophosphate transport regulator (43). Additionally, we noted several genes associated with meat quality (*RPL29*, *ANK1*, and *GSK3A*), production and growth traits (*CDK6*, *ZBTB7C*, *KIF22*, *ATP6V1H*, *UBR2*, and *CUBN*) (44–49).

**Table 1:** Potential selected genes associated with important traits identified by three methods in TRD.

Chr	Position (bp)	Candidate Genes	Traits
4	25,260,001–25,280,000	*MT3*	Skeletal development (50)
4	51,440,001–51,460,000	*GSK3A*	Meat quality and fat deposition (52)
5	93,280,001–93,300,000	*MYO18A*	Immune response (39)
5	100,520,001 –100,540,000	*NDEL1*	Heat adaptability (35)
9	6,800,001–6,820,000	*OBSCN*	Muscle contraction (52)
9	8,240,001 –8,260,000	*UPF1*	Heat adaptability (53)
10	40,280,001–40,300,000	*KIF22*	Skeletal development (44)
11	95,760,001–95,780,000	*IL1R1*	Heat adaptability (51)
14	76,820,001–76,840,000	*F13B*	Heat adaptability (37)
17	19,000,001–19,020,000	*LEF1*	Immune response (54)
18	20,400,001–20,420,000	*CDK6*	Body size (49)
20	31,740,001–31,760,000	*FCER1G*	Immune response (40)
20	101,900,001 –101,920,000	*CACHD1*	Heat adaptability (35)
21	25,620,001–25,640,000	*ATP6V1H*	Skeletal development (48)
23	31,640,001–31,660,000	*CUBN*	Skeletal muscle development (47)
24	23,260,001–23,280,000	*DCLK3*	Heat adaptability (38)
24	25,060,001–25,080,000	*CCR8*	Immune response (55)
24	64,300,001–64,320,000	*CXCR6*	Immune response (42)
24	59,960,001–59,980,000	*RPL29*	Meat quality and fat deposition (56)
25	58,680,001–58,700,000	*ANKH*	Skeletal development (43)
33	55,140,001–55,160,000	*ARHGAP15*	Heat adaptability (36)

The functional enrichment analysis for 573 overlapped genes were further analyzed. The significantly enriched GO terms and KEGG pathways are shown in Supplementary Table S9. Among them, a total of 267 significantly enriched GO terms with *p*-value < 0.05 were observed, such as heat shock protein binding (GO:0031072, *p* = 0.015360), cellular response to oxidative stress (GO:0034599, *p* = 0.004995), response to bacterium (GO:0009617, *p* = 0.021797), regulation of neuron projection development (GO:0010975, *p* = 0.004311), and positive regulation of phagocytosis (GO:0050766, *p* = 0.011622), which may play role in desert adaptation. Several genes, including *FCER1G*, *ALCAM*, *CCR8*, *CXCR6*, *CNTFR*, *TNFRSF13C*, and *IL1R1*, were significantly enriched in the external side of plasma membrane, which is key for environmental signal perception and immune cell communication (GO:0050766, *p* = 0.037169). In addition, metabolism-related and growth-related biological functions were significant, such as regulation of bone mineralization (GO:0030500, *p* = 0.026519), Wnt signaling pathway (GO:0016055, *p* = 0.000777), activation of protein kinase B activity (GO:0032148, *p* = 0.021715), ATP binding (GO:0005524, *p* = 0.000015), and microtubule-based movement (GO:0007018, *p* = 0.048545). *ANKH* and *SGMS2* were both enriched in the regulation of bone mineralization (GO:0030500, *p* = 0.026519), suggesting their potential involvement in antler formation and skeletal development in red deer. *MT3*, which is involved in activation of protein kinase B activity (GO:0032148, *p* = 0.021714), exerts a key influence on osteoclastogenesis and osteoporosis via dual routes involving reactive oxygen species and specificity protein 1 (50). Furthermore, 45 significant enriched pathways were obtained, including MAPK signaling pathway (bta04010, *p* = 0.001910), cell cycle (bta04110, *p* = 0.006348), cytokine-cytokine receptor interaction (bta04060, *p* = 0.027605), and Jak–STAT signaling pathway (bta04630, *p* = 0.005778), which are related to immune response and skeletal development. The enrichment of interleukin-1 receptor activity (GO:0004908, *p* = 0.003269) and MAPK signaling pathway (bta04010, *p* = 0.001910) further supports the role of *IL1R1* as a vital mediator in many immune and inflammatory responses induced by cytokines, which has been reported to be involved in the heat stress response (51). Overall, the results suggest that TRD has experienced strong selection on genes related to immunity, skeletal development, and stress resistance, reflecting a genetic adaptation to arid desert environments.

## 4 Conclusion

The study showed that TRD population has relatively low genetic diversity and a high level of inbreeding. The results also validate the classification of TRD, ERD, and CRD into three distinct branches, with TRD being more closely related to CRD. Additionally, based on whole-genomic data, candidate genes related to adaptation to heat environments were identified and annotated. These findings will be very useful for the future conservation and management of TRD and determining the potential genomic mechanisms in harsh environments.

## Data Availability

The datasets presented in this study can be found in online repositories. The names of the repository/repositories and accession number(s) can be found in the article/Supplementary material.

## Footnotes

^1^ https://www.ncbi.nlm.nih.gov/sra/

^2^ https://github.com/broadinstitute/picard

## Supplementary material

Supplementary Figure S1 The picture of an adult Tahe red deer.
